# Exploiting Clonal Evolution to Improve the Diagnosis and Treatment Efficacy Prediction in Pediatric AML

**DOI:** 10.3390/cancers13091995

**Published:** 2021-04-21

**Authors:** Salvatore Nicola Bertuccio, Laura Anselmi, Riccardo Masetti, Annalisa Lonetti, Sara Cerasi, Sara Polidori, Salvatore Serravalle, Andrea Pession

**Affiliations:** 1 Pediatric Oncology and Hematology “Lalla Seràgnoli”, Pediatric Unit-Department of Medical and Surgical Sciences (DIMEC), University of Bologna, 40126 Bologna, Italy; salvatore.bertuccio2@unibo.it (S.N.B.); laura.anselmi4@unibo.it (L.A.); 2 Pediatric Oncology and Hematology “Lalla Seràgnoli”, Pediatric Unit-IRCCS Azienda Ospedaliero-Universitaria di Bologna, 40138 Bologna, Italy; sara.cerasi@gmail.com (S.C.); sara.polidori3@studio.unibo.it (S.P.); salvatoreserravalle@libero.it (S.S.); andrea.pession@unibo.it (A.P.); 3 Department of Biomedical and Neuromotor Sciences, University of Bologna, 40126 Bologna, Italy; annalisa.lonetti2@unibo.it

**Keywords:** pediatric AML, NGS, clonal evolution, target therapy

## Abstract

**Simple Summary:**

The use of innovative technologies has revolutionized cancer research in recent years, and in the field of pediatric oncohematology, great results have been achieved. However, within this context, acute myeloid leukemia still represents a considerable challenge for clinicians, as frequent cases of relapse or refractory disease, especially in specific subgroups, remain present. With this review, the authors aim to recapitulate the main features of this extremely heterogeneous malignancy, by highlighting the concept of clonal evolution and how, thanks also to the impact of new high throughput techniques, this could be exploited to deepen the current knowledge on molecular mechanisms driving this disease. Overall, this study will seek to pave the way for making new tools available to further improve diagnosis and treatment protocols in pediatric patients.

**Abstract:**

Despite improvements in therapeutic protocols and in risk stratification, acute myeloid leukemia (AML) remains the leading cause of childhood leukemic mortality. Indeed, the overall survival accounts for ~70% but still ~30% of pediatric patients experience relapse, with poor response to conventional chemotherapy. Thus, there is an urgent need to improve diagnosis and treatment efficacy prediction in the context of this disease. Nowadays, in the era of high throughput techniques, AML has emerged as an extremely heterogeneous disease from a genetic point of view. Different subclones characterized by specific molecular profiles display different degrees of susceptibility to conventional treatments. In this review, we describe in detail this genetic heterogeneity of pediatric AML and how it is linked to relapse in terms of clonal evolution. We highlight some innovative tools to characterize minor subclones that could help to enhance diagnosis and a preclinical model suitable for drugs screening. The final ambition of research is represented by targeted therapy, which could improve the prognosis of pediatric AML patients, as well as to limit the side toxicity of current treatments.

## 1. Introduction

Pediatric acute myeloid leukemia (AML), with an incidence of approximately seven occurrences per 1 million children annually [1], still represents a challenge for pediatric oncologists. Even though patient outcome has significantly improved over the past 30 years [1], as survival rates have reached ~70%, still about 30% of children with AML experience disease recurrence, with a probability of overall survival (pOS) ranging between 29 and 38% [2,3,4,5]. Thus, given the high frequency of relapse in pediatric AML, a deeper understanding of the mechanisms underlying disease recurrence is necessary.

### 1.1. Pediatric AML: Clinical Presentation

AML is a heterogeneous disease, from clinical behavior to morphology, immunophenotyping and genetic abnormalities, due to the abnormal proliferation of myeloid progenitor cells in the bone marrow [6]. It has some unique presentations, such as granulocytic sarcomas, subcutaneous nodules, infiltration of the gingiva and disseminated intravascular coagulation (DIC) [7]. Diagnosis is based on leukemic blasts evaluation from the bone marrow, considering the morphology, cytochemistry, immunophenotype, cytogenetics (conventional karyotyping complemented with FISH or reverse transcriptase PCR) and molecular characterization [6]. AML is then classified according to the 2016 World Health Organization (WHO) classification, which also considers karyotype and molecular aberrations, and has replaced the morphology-based French–American–British (FAB) classification [6]. Due to the poor outcome of some specific subtypes, risk-group stratification based on genetic abnormalities and response is essential, mainly in order to identify those with high-risk AML, which could benefit from a more intensive treatment [1] such as hematopoietic stem cell transplantation (HSCT) in first complete remission (CR) [8]. Despite an increasing knowledge about the landscape of molecular alterations in AML, there are still only few alternatives to standard intensive chemotherapy regimens used for induction and consolidation treatment protocols, considering that ~30% of the pediatric patients relapse after the achievement of CR. The development of new therapies able to overcome resistance/relapse is essential. To this aim, it is necessary to deeply characterize the mechanisms responsible for AML and progression. 

### 1.2. Pediatric AML: Common Genetic Lesions

**Figure 1:**
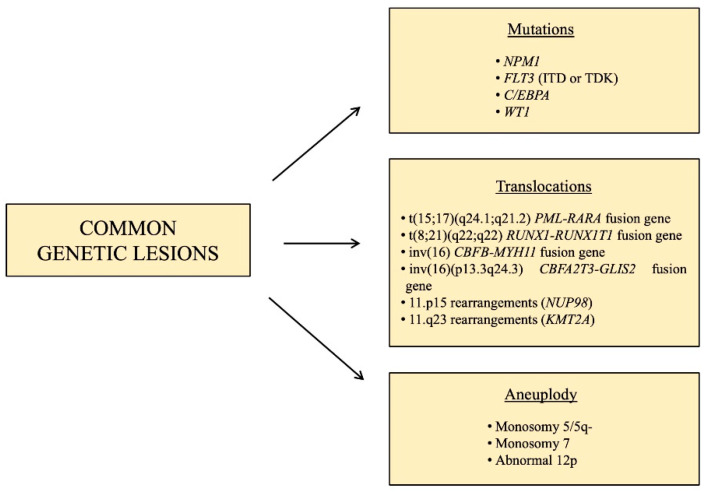
Schematic resume of the main recurrent genetic lesions found in pediatric AML.

Regardless of its heterogeneity, some characteristic chromosomal changes and molecular lesions are recurrent in pediatric AML (Figure 1). These include the translocation t(15;17)(q24.1;q21.2), that leads to the fusion of promyelocytic leukemia (*PML*) gene with the retinoic acid receptor alpha (*RARA*), in acute promyelocytic leukemia, which represent ~5% of pediatric AML [9]. This subtype of AML is highly peculiar and does not belong to any additional category, and its remission rates are >95% and overall survival is >80% [10], thanks to a target therapy consisting of all-trans-retinoic acid (ATRA) associated with arsenic trioxide (ATO), with the addition of the anti-CD33 gemtuzumabozogamicin in high-risk patients, defined as having WBC prior to treatment ≥10 × 109/L [9].

Other subtypes of pediatric AML, characterized by a favorable prognosis, are those involving core binding factor (CBF), accounting for approximately 20–25% of pediatric AML cases [11], such as t(8;21) (q22;q22) and inv(16),which lead to the fusion genes *RUNX1-RUNX1T1* and *CBFB-MYH11,* respectively.

*KMT2A* is a histone methyltransferase that is rearranged in 35–60% of infants and 10–15% of children and adolescents [12], and it has almost 100 different fusion partners [13]. It represents a subgroup of pediatric AML with intermediate/unfavorable prognosis and for this reason, many specific drugs are in preclinical and clinical trials [14]. A subtype of pediatric AML with an unfavorable prognosis is that characterized by the fusion gene *CBFA2T3-GLIS2*, caused by inv(16) (p13.3q24.3), which is present in 15–20% of pediatric non-Down syndrome acute megakaryoblastic leukemia (non-DS-AMKL) and about 7–8% of pediatric AML with normal karyotype [15,16], mostly found in infant patients (<3 years) [17,18]. AMKL occurs particularly in children with Down syndrome, however, in contrast to DS-AMKL which display 80% of overall survival rate, non-DS-AMKL is associated with extremely poor prognosis [19,20]. Fusions involving the gene nucleoporin 98kD (*NUP98*) and over 30 different fusion partners, the most common of which are *KDM5A* and *NDS1*, can be found in 4–9% of pediatric AML [21]. The t(6;9) (p22;q34) leads to the expression of the fusion gene *DEK-NUP214*, present in just 1–2% of pediatric AML [22], whose role in leukemogenesis is unknown, but it is associated with a poor prognosis, due to the low rates of remission and high rates of relapse. A very rare cause of pediatric AML (less than 1%) associated with grim prognosis are translocations or inversions involving the *MDS1* and *EVI1* complex locus (*MECOM*) [13]. Aneuploidy, in the form of monosomy 5/5q-, monosomy 7 and abnormal 12p, are present in 4–9% of pediatric AML [12].

With the advent of large-scale genomic approaches, several molecular aberrations have been likewise identified, such as the gene nucleophosmin 1 (*NPM1*) which encodes for a chaperon protein normally localized predominantly in the nucleolus, however, when mutated, it is aberrantly localized in the cytoplasm, causing the activation of oncogenes. This is rarely altered in pediatric AML [23], but much more often in children (10%) and adolescents (20%) [12], and it often co-exists with *FLT3*-ITD mutations [24]. Another recurrent mutation in AML, especially in older children and adolescents, is that involving the gene CCAAT-enhancer binding protein alpha (*C/EBPA*), present in 5–10% of pediatric AML cases [12]. This is a very important transcription factor for granulocytic and monocytic differentiation [25], and inactivating mutations causing a block in granulocytic differentiation likely contribute to leukemogenesis. The prognosis of this genetic aberration in pediatric AML is still a matter of debate but generally, patients harboring this mutation are included in low/intermediate risk groups. Among the molecular lesions with an unfavorable prognosis, FMS-like tyrosine kinase 3 (*FLT3*) is a common gene involved in pediatric AML, whose frequency is age-related, being rare in infants and rising as age increases. *FLT3* is essential for the proliferation, survival and differentiation of stem/progenitor cells. The most common type of mutation in *FLT3* is represented by internal tandem duplication (ITD) mutations, but point mutations are also frequent, and both these lesions lead to the constitutive activation of this gene [26]. The *WT1* gene is expressed in CD34+ hematopoietic stem progenitors and is crucial for the regulation of normal growth and development. Among pediatric AML cases, 15% are characterized by inactivating mutations of *WT1* [21], and associated with a poor prognosis when *FLT3*-ITD mutations are also present [21]. Mutations in epigenetic regulators such as *TET2*, *IDH1* and *IDH2* are much less prevalent compared to adult AML, characterizing only 1–2% of pediatric patients [27], while signaling mutations such as *NRAS*, *KRAS*, *CBL*, *GATA2*, *SETD2* and *PTPN11* are more common in younger patients. Moreover, MYC alterations were identified as exclusive in children, suggesting different leukemogenesis mechanisms in children compared to adults [10,21].Nonetheless, therapies targeting alterations in these factors are showing promising results in adults, thus it is important to look for these lesions in pediatric cases too [27]. 

### 1.3. Clonal Evolution in Pediatric AML: From Diagnosis to Relapse

The concept of clonal evolution in cancer, which was first conceived in 1976 [28], appears to apply also to AML, as demonstrated by the pivotal work of Ding et al. [29] in adult AML and also confirmed in the pediatric setting [2,3,4,5,6,7,8,9,10,11,12,13,14,15,16,17,18,19,20,21,22,23,24,25,26,27,28,29,30]. This term refers to the selection and expansion, over the course of the disease, of subclones that originate from a common ancestral clone but acquire different mutations that confer them a survival advantage, leading to genetic diversity within a cell lineage [2]. AML arises from the accumulation of “early” DNA mutations in hematopoietic stem cells (HSCs), which enhance their self-renewal potential, leading to leukemia stem cells (LSCs). Additionally, “late” mutations that promote proliferation are necessary for the development of AML [31]. We can therefore identify “primary events” that are highly penetrant and stable during the course of the disease, and “secondary events” that occur later and only in some cells, leading to the development of different subclones and to a variation in the composition of cancer [2]. This explains the complexity of the determination of the proper treatment option, because of AML genomic heterogeneity not only at diagnosis, but also during therapy and eventually at relapse. 

Several studies [2,30,32] have globally analyzed, by means of whole exome sequencing (WES), trios of pediatric AML diagnostic, remission and relapse specimens, in order to deeply characterize the key features that shapes clonal evolution over time. They showed that most of the dominant variants, which originate during ancestral leukemic development, persist from diagnosis to relapse, but many subclonal modifications, which develop later in specific subclones, do not, and thus, the genomic landscape at relapse also differs based on the use of different therapeutic agents [30]. They also demonstrated that, even if recurrent single mutations cannot be found, mutations in specific gene families are typical [30]. Indeed, aside from the canonical classification of AML, in terms of clonal evolution, we can further classify mutations into three major groups (Table 1):

i. Cohesin complex gene mutations;
ii. Transcription factors and epigenetic regulators mutations;
iii. Signaling molecules mutations.

Cohesin is a protein necessary for cell division, DNA repair and gene expression, and it is composed of four core subunits, three of which have been found to be mutated in pediatric AML, leading to a loss of cohesin function: *RAD21*, *SMC3* and *STAG2* [32]. *Shiba* et al. [32] demonstrated that variant allele frequencies (VAFs) of mutated *SMC3* were high, suggesting these occur at an early stage of leukemogenesis. 

Among the epigenetic regulators, mutations have been found in the Additional Sex combs-Like (*ASXL)* family, *BCOR* and *BCORL1*, *EZH2*. *ASXL1* and *ASXL2* mutations have been found in pediatric de novo AML, mainly in patients with t(8;21) [32], and an *ASXL3* mutation has been described by *Masetti* et al. [2] at relapse (median frequency or MF of 29.7%), backtracking to a small subclone already present at diagnosis (MF of 0.3%). VAFs of mutated *ASXL2* were lower than others, suggesting that this mutation is a secondary event. *BCOR* and its homologue *BCORL1* suppress gene transcription through epigenetic mechanisms [32]. *BCORL1* has been shown to be a tumor suppressor gene, and, as *SMC3*, *BCORL1* appears to be mutated at an early stage in leukemic cells [32]. *EZH2* is one of the most commonly mutated genes in pediatric Down syndrome AMKL, and it encodes for a subunit of a complex responsible for methylation [32]. 

Signaling pathway mutations include mutations of genes involved in Ras pathways such as *NRAS*, *KRAS*, *PTPN11*, and of tyrosine kinases such as *KIT* and *FLT3*. *NRAS* mutations have been found to be relapse specific, like those in *CREBBP*, a coactivator of several hematopoietic transcription factors [32]. *Farrar* et al. [30] described *PTPN11* mutations, lost at relapse when they appeared as subclonal variants at diagnosis. *Masetti* et al. [2] found a *PTPN11* mutation gained at relapse (MF of 31.9%), suggesting its role in increasing cell proliferation and/or survival. As previously described, *FLT3* is essential for proliferation, survival and differentiation of stem/progenitor cells. *Masetti* et al. [2] showed that a small *FLT3-TKD*-mutated subclone present at diagnosis (MF of 3.4%) underwent expansion at relapse (MF of 13.3%), leading to increased cell proliferation and/or survival, and this mutation, together with *NRAS* and *KIT* mutations, was also described as a secondary event contributing to disease progression by *Shiba* et al. [32]. 

In addition, also genes that do not belong to these groups are frequently mutated. The previously described *C/EBPA* and *WT1* mutations exhibit different patterns of recurrency in the study presented by *Masetti* et al. A highly penetrant biallelic mutation of *C/EBPA* was revealed, and in one patient, a homozygous non-frameshift insertion was present both at diagnosis and relapse in the majority of the tumor-cell population (MF > 80%). On the contrary, *WT1* mutations appeared highly unstable, two different frameshift insertions were detected mainly at diagnosis (MF of 27.6% and 13.9), while a single-nucleotide variant was detected only at relapse (MF of 40%). *SETD2* is a methyltransferase involved in the recruitment of mismatch repair (MMR) machinery, and its mutation results in a loss of function of the methyltransferase activity, leading to the build-up of several subclonal mutations and consequently, the increased plasticity and adaptability of leukemia cells, due to the failure of DNA repair [2]. A frameshift insertion of the *SETD2* gene has been described both at diagnosis (MF of 32.5%) and at relapse (MF of 31.7%) [2]. The *TYK2* gene is a member of the Janus tyrosine kinases (JAK) family involved in cell growth, differentiation and survival. *Masetti* et al. [2] found a mutation of *TYK2* both at diagnosis (MF of 43%) and at relapse (MF of 14.9%), which causes a hyperactivation of the *TYK2* pathway, resulting in aberrant cell survival through the upregulation of BCL2 (anti-apoptotic protein). *SALL1* is a member of the transcriptional network that regulates stem cell pluripotency [33]; it has not been detected at diagnosis but only at relapse (MF of 28.6%) with a clone size at relapse of 50–60% (corrected for copy number variations) [2] (Table 1).

## 2. Identifying Genetic Heterogeneity and Subclones Populations Exploiting High Throughput Techniques

Even though limited literature is available in the pediatric context regarding clonal evolution studies, massive sequencing has already been performed on multiple pediatric leukemia patients samples, taken both at diagnosis and relapse, that allowed the broad characterization of major genetic lesions and subclonal leukemic populations, as described above [2,34]. However, additional considerations need to be taken into account and further progress, helpful in translating these data to improve clinical settings and evaluations, could be achieved by means of innovative technical approaches. Recent progresses in microfluidics and molecular barcoding have supported the extraordinary expansion of single-cell analysis, which represents a remarkable opportunity to finely characterize DNA, RNA and proteins at single-cell resolution [35]. As experimental protocols have become more accessible for large-scale samples analysis, a huge variety of information could be exploited for research purposes, but also as a powerful technology to help diagnostic evaluations in the clinic, by identifying the complex genetic heterogeneity of leukemic cells population through the time and their deregulated druggable weaknesses. We will present herein interesting innovative studies even if mostly based on the adult leukemic background, as we consider these original works fundamental to suggest novel approaches to diagnose and treat pediatric acute leukemias as well. For instance, *van Galen* et al. refined and established a short-read and nanopore sequencing method to detect genetic aberrations in individual cells [36]. Eventually, thanks to a machine learning classifier, they merged the data coming from both transcriptional and genetic analysis to describe six malignant cell types and distinguish them from healthy samples. Thus, this also suggests a way to evince pre-malignant clones and their evolution through disease progression. Furthermore, in this study, they took advantage of single-cell profiling also to notice the functional differences in *FLT3* genotypes within the same patient. For instance, an in-depth analysis revealed that four different *FLT3* alleles co-exist and they were referred to distinct subclones; the one with *FLT3*-ITD contained more primitive cells, while another with *FLT3*-TKD contained more differentiated cells. These variations might explain how AML differentiation is affected and likewise why it is correlated with a poor outcome [36,37].

A recently published study by *Morita* et al. introduced multiple single-cell genomics techniques to reveal the molecular architecture of a panel of 123 AML patients [38]. Single-cell DNA sequencing (scDNA-seq) identified major somatic mutations with relative zygosity state, which were then validated either by bulk-seq, droplet digital PCR or quantitative PCR assay. These include the well-known *NPM1*, *DNMT3A*, *FLT3*, *NRAS*, *KRAS*, *SRSF2* and *TET2*. The pair-wise analysis of mutation co-occurrence using single-cell data provided evidence of specific clonal relationship of AML driver mutations. Afterwards, they took advantage of a probabilistic model, single cell inference of tumor evolution (SCITE), to generate a phylogenic tree that recapitulated the evolutionary story of AML cells population [39]. To further characterize the leukemia initiating cells (LICs), different samples with a highly branching clonal structure were xenotransplanted in immunodeficient mice and tested for their ability to engraft (CD34+ cells analyzed in scDNA-seq). Most of the subclones previously identified were found in the engrafted sample, and they showed a different capacity to regenerate. For instance, in one of the AML samples, two distinct subclones were carrying a similar panel of mutations, except for a point mutation in *RAS* (*NRAS* p.G12S or *KRAS* p.Q61H). In a PDX model derived from this sample, there was a considerably major expansion of the clone *NRAS* p.G12S. Interestingly, the same clonal evolution was observed in that specific AML patient after therapy, thus suggesting an important role for PDX in vivo studies, which reflects the actual assortment of leukemic cells [38]. 

Moreover, another fundamental tool is represented by immunophenotypic analysis, performed in the aforementioned study by scDNA + protein-seq and then validated in multi-color flow cytometry. There is a clear association between some mutations and the expression of specific surface markers, for instance *NPM1* associated with lower CD34 expression, whereas *TP53* with higher CD34 expression [38]. The use of combined data from mutational analysis and immunophenotyping is able to give an evolutional portrait of the different subclones. For instance, preleukemic mutations are correlated with both myeloid and lymphoid markers, while the acquisition of driver mutations are more associated with early progenitors stemness markers (e.g., CD34, CD117) [38]. An additional example is represented by a case study of a 7-year-old patient diagnosed AML with complex karyotype, wild-type *FLT3*, negative for common chromosomal inversions or translocations, in which *Pereira* et al. analyzed the immunophenotyping evolution from diagnosis until a third relapse by means of multiparametric flow cytometry [40]. At diagnosis, there were two distinct populations, one expressing CD34/CD33/CD117/CD11b/CD56 and the other with the same markers with additional CD7 expression. After the first relapse, three subclones emerged, one that persisted from the diagnosis and the other two with high or low level of CD19, respectively, but this time all clones were CD7 positive. Even after bone marrow transplantation, a second relapse eventually occurred, with all blasts presenting CD19 but divided in two distinct populations either with or without CD56 and CD7. Despite further chemotherapy, a persistent disease was present, expressing CD7/CD56/CD19 and CD3, with low levels of myeloperoxidase although the original myeloid markers were maintained [40]. Interestingly, this study highlighted the importance of multiparametric flow cytometry in defining subpopulations during the clonal evolution of the disease, in which some populations disappeared while others persisted and gave birth to new subclones in the subsequent relapses. 

In addition, deep sequencing and new tools for analysis can help find predictive models of acute myeloid leukemia risk in healthy individuals, as demonstrated by the work of *Abelson* et al. [41] in the adult context. Peripheral blood cells from 95 individuals obtained roughly 6 years before AML diagnosis (pre-AML) were analyzed together with 414 random individuals (control group). The pre-AML group was more prone to genetic aberrations (in particular in a panel of specific genes) and higher variant allele frequencies, sign of a greater clonal expansion. Indeed, they deduced that on one side, basic clinic and laboratory information are able to identify high-risk subgroup months before AML diagnosis; however, thanks to deep genetic analysis, these data could identify cases even several years before the presentation of the disease [41]. The timeline of accumulation of somatic mutations and their clonal ramification has already revealed its importance in the understanding of leukemogenesis but also in the choice of the proper treatment and diagnosis. Nonetheless, discerning how these mechanisms occur is also of fundamental importance to develop strategies to predict and prevent disease progression. Indeed, some mutations, particularly in the genes involved in DNA repair (*FANC* genes), telomere biology disorder (*CTC1*, *RTEL1*, *WRAP53*), germline myeloid neoplasm-associated genes (*DDX41*, *RUNX1*) and others such as *C/EBPA* and *WT1*, are associated with a predisposition to an earlier onset of AML and cases of familial AML [42,43,44]. One of the most interesting ways to apply clonal evolution studies and high throughput technologies to the clinic is represented by the future perspective to be able to investigate AML response to different treatments, in particular by analyzing those clones emerging after therapy, which are often involved in relapse. The use of high throughput analysis in single cells will allow a much deeper comprehension of clonal evolution and diversity, and, most of all, of key leukemic drivers that are yet not considered, but also potential germline variants, thus improving the predictive and prognostic considerations of AML cases.

## 3. iPSC Model as a Promising Tool to Study the Clonal Evolution of AML

As previously described, pediatric AML, both at diagnosis and relapse, is characterized by a prominent clone but also by minor subclones. As a consequence, molecular heterogeneity corresponds to different levels of sensitivity to conventional therapies. However, nowadays, it is not always possible to easily handle enough patients’ primary blasts to perform comprehensive studies, thus motivating the development other innovative cells systems to do so. In the last 10 years, the advent of induced pluripotent stem cells (iPSCs) has offered a unique tool to isolate different subclones by reprogramming patients’ specific iPSCs. In this context, the reprogramming process of AML blasts can allow to isolate single clones that are useful to test different drugs. Indeed, for this purpose, *Chao* et al. reprogrammed AML adult blasts with MLL rearrangement [45]. The target screening of iPSC colonies brought out a small subclone with *MLL-r* and *KRAS* mutation. The hematopoietic cells iPSC-derived with *KRAS* and *MLL-r* had different biological properties in terms of engraftment in NSG mice and, above all, in terms of response to different drugs. Indeed, the proliferation of this subclone is affected by means of a KRAS inhibitor as well as by ARA-c. The retrospective target screening of a patient’s blasts elucidated that at diagnosis, the *KRAS* clone was predominant, but it was not detected at relapse. This was in agreement with the iPSC model, in which hematopoietic cells with *KRAS* mutation were responsive to ARA-c, while the hematopoietic cells with *MLL*-r and *RAS* wild type were resistant to chemotherapy. Moreover, the hematopoietic differentiation of iPSCs derived from AML patients confirmed that these cells clustered in a different way in terms of gene expression and methylation, compared to the hematopoietic cells derived from control iPSCs, thus supporting the fact that the mutations present in AML-iPSC drive the leukemic phenotype only upon differentiation to hematopoietic cells (and not to other differentiated tissues) [45]. 

The use of the iPSCs model to study clonal evolution can also be found in the research of *Kotini* et al. [46]. By reprogramming different kinds of patients’ samples, ranging from the preleukemic state to secondary AML, they were able to characterize the specific stages and features of derived cells, thus enabling a broad description of disease progression culminating in serially transplantable leukemia. Even more importantly, they were able to shape these key characteristics by means of CRISPR/Cas9-mediated genetic modifications, in order to describe how this not only affects cells’ stage-specific features, but also their response to distinct drugs [46]. 

On the other hand, the improvement in genome editing knowledge allowed the possibility to engineer iPSCs derived from a healthy donor with onco-fusion genes. In this context, *Bertuccio* et al. used a knock-in healthy donor iPSC with *ETO2-GLIS2* under the control of a pan hematopoietic promotor [47]. The differentiation of these iPSCs into hematopoietic lineage revealed that hematopoietic cells reproduced the cellular and molecular features of non-DS-AMKL pediatric patients.

## 4. Conclusions and Future Perspective

The concept of clonal evolution has impacted in several ways the research approach in studying hematological diseases. The idea of a concrete cells’ heterogeneity, finely modulated at both chromatin, gene and protein level, that is able to give rise to specific paths of disease progression, has displaced the focus to the necessity of new methods to reach a deeper knowledge on how to treat a specific disease. Notably, this is an urgent need in pediatric hematological malignancies, in which, even if the treatment and survival rates have reached significant results, still frequent unclear cases of relapses and drug resistance are present. As previously reported, a huge amount of work lead to the discovery of most of the prominent genetic lesions in pediatric acute leukemias, and advanced high throughput technologies were fundamental to achieve this goal. Many innovative research works and tools, even if mostly considering adult samples, indeed highlighted the importance of different subclones in tuning AML from a preleukemic state to the overt disease. Furthermore, being able to identify and classify these subclones is also an essential issue to examine treatment responses, as different cell populations have exhibited a different degree of sensitivity and some of them might be the ones responsible for therapy resistance cases. In this context, the adoption of iPSC models in the research field has allowed to add a further means to unravel obscure mechanisms in clonal evolution and leukemic progression. Furthermore, this model is even more relevant for the pediatric cause, as it provides a way to analyze the whole process starting from embryonic-like cells development. The molecular mechanisms through which specific genetic lesions give rise to specific phenotypes (i.e., a particular FAB subtype of AML rather than others, or the fact that they affect only myeloid progenitors rather than lymphoid, even in the case of widely spread oncogenic aberration) often take place in an early phase of differentiation, and stage-specific alterations contribute to distinct outcomes. Additional studies are needed to deepen the understanding of such mechanisms, which are of vital importance to immediately identify the disease at the diagnostic level, but also to precisely determine the proper effective treatment at clinical level. The future is, and has to be, oriented towards targeted therapy and tools that will help the clinics to easily manage pediatric patients, in order to help those cases which have to date been so difficult to interpret and to cure.

## Figures and Tables

**Table 1:** List of the main mutated genes, classified in three major groups, with the relative prominent occurrence in terms of timing (mainly at diagnosis and/or relapse).

Group of Mutations	Genes	Timing
Cohesin complex genes	*RAD21, SMC3, STAG2*	Diagnosis
	*ASXL1, ASXL2*	Diagnosis
	*ASXL3*	Relapse
	*BCOR, BCORL1, EZH2*	Diagnosis
Signaling molecules	*NRAS, CREBBP, KIT, FLT3-ITD*	Relapse
	*PTPN11*	Diagnosis/relapse
Others	*SETD2, TYK2*	Diagnosis/relapse
	*SALL1*	Relapse
	*C/EBPA*	Diagnosis
	*WT1*	Diagnosis/relapse
